# Histological Diversity of Eyelids and the Nictitating Membrane in Six Woodpecker Species (Picidae)

**DOI:** 10.3390/vetsci13070702

**Published:** 2026-07-17

**Authors:** Joanna Klećkowska-Nawrot, Aleksandra Kroczak-Zdańkowska, Adam Urantówka, Grzegorz Zaniewicz, Aleksander Chrószcz, Dominik Poradowski

**Affiliations:** 1 Department of Biostructure and Animal Physiology, Division of Animal Anatomy, Faculty of Veterinary Medicine, Wrocław University of Environmental and Life Sciences, Kożuchowska 1, 51-631 Wrocław, Poland; 2 Department of Genetics, Faculty of Biology and Animal Science, Wrocław University of Environmental and Life Sciences, Kożuchowska 7, 51-631 Wrocław, Poland; 3 Ecophysiology and Behavioral Ecology Research Unit, Department of Vertebrate Ecology and Zoology, Faculty of Biology, University of Gdańsk, Wita Stwosza 59, 80-308 Gdańsk, Poland

**Keywords:** Piciformes, Picidae, eyelids, nictitating membrane, CALT, histology

## Abstract

Woodpeckers (Picidae) are exposed to repeated mechanical loading and particulate material during climbing, drilling, pecking, and foraging on tree trunks, but the microscopic organization of their eyelids and nictitating membrane remains poorly known. This study asked whether European woodpeckers share a common histological pattern of ocular adnexa and whether selected structural features may be interpreted in the context of ocular surface protection. The upper eyelid, lower eyelid, and nictitating membrane were examined in 40 birds representing six European woodpecker species using routine histological and histochemical methods. In all of these species, the eyelids showed a layered organization with a keratinized anterior palpebral surface, non-keratinized conjunctival surface with goblet cells, marginal zone, musculofibrous stroma, and no tarsal glands. Species-related differences were observed in the examined material in epithelial thickness, skin fold development, melanin pigment distribution, and marginal zone organization. The lower eyelid contained a compact fibrous tarsal plate and CALT represented by lymphoid nodules and diffuse lymphocytes, with morphologically identified HEV-like venules located within or close to lymphoid tissue. The nictitating membrane showed regional variation in the marginal plait, leading edge, epithelial surfaces, stromal pigmentation, and fold organization. These findings provide the first comparative histological baseline for the eyelids and the nictitating membrane of European Picidae and support the cautious interpretation that these structures may contribute to mechanical, mucosal, and immune protection of the ocular surface in woodpeckers. Because sample sizes were unequal, these interspecific observations should be treated as descriptive, sample-level findings rather than definitive species-level traits.

## Simple Summary

Woodpeckers are birds adapted to climbing, foraging on tree trunks, drilling, and pecking. During these activities, the eye region may be exposed to small wood particles, bark fragments, dust, and repeated mechanical irritation. For this reason, the eyelids and nictitating membrane may play an important protective role. In this study, we examined the upper eyelid, lower eyelid, and nictitating membrane in six European woodpecker species. The material was collected *post-mortem* from birds found dead in their natural environment. We described the microscopic structure of the eyelids and nictitating membrane, including the epithelium, connective tissue, muscle bundles, blood vessels, pigment cells, goblet cells, and lymphoid tissue. In all the examined species, the eyelids had a similar general organization, but some differences were observed between species. These differences concerned epithelial thickness, skin folds, melanin pigmentation, and the structure of the nictitating membrane. A relevant observation in this species group was the documentation of conjunctiva-associated lymphoid tissue in the lower eyelid. This tissue may participate in local immune protection of the ocular surface, but its importance is interpreted in the context of Picidae rather than as a novel feature of birds in general. The results provide basic comparative data on the eyelids and the nictitating membrane in woodpeckers and may be useful for future studies of avian ocular anatomy.

## 1. Introduction

Birds have an upper eyelid (palpebra dorsalis), a lower eyelid (palpebra ventralis), and a nictitating membrane (palpebra tertia) [1]. These structures protect the ocular surface, contribute to the distribution and stability of the precorneal tear film, and support normal vision under different environmental conditions [2,3]. In most birds, the upper eyelid is relatively short and thick, whereas the lower eyelid is longer, thinner, and more mobile. The lower eyelid usually provides most of the corneal coverage during eyelid closure, although the relative development and mobility of the eyelids may vary among avian groups [2,4,5,6]. The upper and lower eyelids are composed of three main layers: a superficial skin layer, a middle musculofibrous layer, and an inner conjunctival layer. The conjunctiva is lined by epithelium with goblet cells and contains connective tissue that may include diffuse or organized lymphoid tissue [7,8].

The nictitating membrane is a well-developed component of the avian ocular adnexa. Unlike the upper and lower eyelids, it is moved mainly by the quadratus and pyramidal muscles [1,4,8]. During movement over the cornea, the nictitating membrane helps distribute the precorneal tear film, remove small particles from the ocular surface, and provide additional mechanical protection without complete closure of the palpebral fissure [9]. Its free margin and epithelial surface may show folds or papillary specializations involved in tear distribution and clearance of debris [7,8,10,11]. Because the nictitating membrane moves directly across the corneal surface, its epithelial and stromal organization may be particularly relevant in birds exposed to dust, particulate matter, or repeated mechanical irritation of the periocular region.

Protection of the avian ocular surface also involves local immune mechanisms. In birds, conjunctiva-associated lymphoid tissue (CALT) and the Harderian gland are considered important components of ocular mucosal immunity [3,12,13]. CALT may occur as diffuse lymphocytes in the conjunctival *substantia propria* or as organized lymphoid nodules/follicles located beneath the conjunctival epithelium [7,10,11,14,15]. In the chicken, organized CALT has been described particularly in the lower eyelid, where lymphoid follicles contain high endothelial venules (HEVs), which are associated with lymphocyte migration into the tissue [13,14,15]. Although CALT is not unique to woodpeckers and has been reported in several avian species, its distribution and organization remain poorly documented in many wild bird groups, including Picidae. Therefore, describing CALT in woodpeckers is relevant as part of a broader comparative assessment of ocular surface defense rather than as evidence of a woodpecker-specific immune structure.

Woodpeckers (Picidae) represent one of the most specialized avian lineages in terms of cranial biomechanics. Repeated pecking produces rapid head accelerations and exposes the orbital region to wood particles, bark fragments, dust, sap, and other environmental material [16,17,18,19]. While adaptations of the skull, hyoid apparatus, cervical musculature, and ocular protection during pecking have been discussed previously, the eyelids and nictitating membrane remain poorly characterized at the microscopic level. This represents an important gap because these structures are directly involved in ocular surface protection, precorneal tear film distribution, removal of particulate material, and local mucosal defense. In woodpeckers, these functions may be especially relevant during climbing, drilling, pecking, and foraging on tree trunks, when the periocular region is repeatedly exposed to wood dust, bark fragments, sap, soil particles, and mechanical vibration.

The central research question of this study was whether European woodpeckers share a common histological organization of the upper eyelid, lower eyelid, and nictitating membrane, and whether selected structural differences among species may be interpreted in the context of ocular surface protection. Therefore, the aim of this study was to provide the first comparative histological description of the eyelids and nictitating membrane in six European woodpecker species: the Gray-headed Woodpecker (*Picus canus*), European Green Woodpecker (*Picus viridis*), Black Woodpecker (*Dryocopus martius*), Great Spotted Woodpecker (*Dendrocopos major*), Middle Spotted Woodpecker (*Dendrocoptes medius*), and Lesser Spotted Woodpecker (*Dryobates minor*). The selected species cover a broad range of morphological and body-size variation within European Picidae, allowing a descriptive comparison across a representative spectrum of European woodpecker forms. The analysis focused on epithelial organization, connective tissue support, pigmentation, musculofibrous and vascular components, the lower-eyelid tarsal plate, CALT, and the regional organization of the nictitating membrane.

## 2. Materials and Methods

### 2.1. Animals and Sample Collection

The study was performed on 40 woodpeckers representing six European species within the family Picidae (order Piciformes): the Gray-headed Woodpecker (*Picus canus*), European Green Woodpecker (*Picus viridis*), Black Woodpecker (*Dryocopus martius*), Great Spotted Woodpecker (*Dendrocopos major*), Middle Spotted Woodpecker (*Dendrocoptes medius*), and Lesser Spotted Woodpecker (*Dryobates minor*). All specimens originated from forest and parkland habitats, where they were found dead in their natural environment between 2020 and 2023. No birds were killed, captured, or handled for the purposes of this study. After collection, samples were stored at approximately −18 °C to −20 °C, and tissue preservation was evaluated during dissection and preliminary histological examination. Individuals were classified into juvenile, immature, or adult age classes based on plumage characteristics according to standard criteria [20,21,22]. Because morphological and gonadal sex determination was not always feasible, particularly in juveniles, final sex identification was performed using molecular methods. Molecular sexing was conducted according to the protocol described by Klećkowska-Nawrot et al. [23] for the Great Spotted Woodpecker. Relevant DNA fragments were amplified using the reaction mixture and CHD1iA amplification protocol according to Kroczak et al. [24]. The amplification results are shown in the Supplementary Material (Figure S1). The complete dataset, including species names, sample sizes, sex and age classes, is summarized in Table 1. Because all examined species belong to the same avian family, habitat data served purely as biological context rather than a basis for formal ecological or phylogenetic comparisons. Throughout the manuscript and figure legends, the examined species are presented in a fixed taxonomic sequence following AviList [25] to facilitate direct comparisons.

For biological context, the examined taxa cover a broad body-size gradient within European Picidae. Based on published species accounts, approximate adult body length and body mass ranges are as follows: *Dryobates minor*, 14–16.5 cm and 17–25 g; *Dendrocoptes medius*, 20–22 cm and 50–85 g; *Dendrocopos major*, 20–24 cm and 70–100 g; *Picus canus*, 25–28 cm and 125–165 g; *Picus viridis*, 30–36 cm and 138–250 g; and *Dryocopus martius*, 45–57 cm and 250–370 g [26,27,28,29,30,31]. Direct measurements of eye size, palpebral fissure length, and head size were not possible in a standardized manner because the material originated from field-found carcasses with variable preservation of the periocular region; therefore, these variables were not included in the comparative analysis.

### 2.2. Macroscopic Examination and Tissue Sampling

Both orbital regions were dissected from each bird whenever the condition of the carcass allowed further examination. The upper eyelid, lower eyelid, and nictitating membrane were examined macroscopically using a Zeiss Stemi 2000-C stereomicroscope (Carl Zeiss, Jena, Germany). The examination included the general preservation of the periocular region, the position and external appearance of the eyelids, and the visibility and condition of the nictitating membrane. Photographic documentation of the dissected material was obtained using a Zeiss Stemi 2000-C stereomicroscope equipped with a Canon EOS 300D digital camera (Canon Inc., Tokyo, Japan). The upper eyelid, lower eyelid, and nictitating membrane were collected from the left and right orbital region, depending on the preservation of the material. The upper and lower eyelids were sampled to include the external skin surface, musculofibrous layer, and palpebral conjunctiva. Sampling was standardized at the anatomical-structure level rather than at a strictly fixed medial-central-lateral point. Where preservation permitted, sections were selected to include the eyelid margin together with the adjacent anterior and posterior palpebral surfaces; the central part of the eyelid was preferred. In field-collected *post-mortem* material, however, some samples were available only from the best-preserved intact portion of the eyelid, and the exact medial (rostral), central, or lateral (caudal) origin could not always be assigned with certainty. Therefore, regional position within the eyelid was not used as a comparative variable. The nictitating membrane was sampled as completely as possible, including the marginal plait, leading edge, palpebral surface, bulbar surface, and stromal component, whenever these structures were preserved. Samples showing advanced autolysis, marked mechanical damage, severe dehydration, fragmentation, or indistinct anatomical boundaries were excluded from microscopic evaluation. Because the material was obtained from birds found dead in the field, the quality of tissue preservation differed among specimens and species. Therefore, all samples were first assessed macroscopically and then screened histologically using Mayer’s hematoxylin and eosin staining. Only samples in which the epithelial and connective tissue, as well as vascular, muscular, and lymphoid components were sufficiently preserved were included in the final histological description.

### 2.3. Ethical Statement

All study material was obtained *post*-*mortem.* No birds were killed, captured, handled, or experimented upon for this research. Consequently, ethical approval was not required under the Polish Act of 15 January 2015 (Dz.U. 2015, item 266, as amended) or Directive 2010/63/EU. Sample collection, possession, transfer, and storage were authorized by the Regional Directors for Environmental Protection in Wrocław (WPN.6401.83.2021.MH) and Gdańsk (WZG.6401.248.2020.AN.2), and the District Veterinary Officer in Wrocław (PU.555.15.2020). Permit copies are available from the corresponding author upon reasonable request.

### 2.4. Tissue Processing and Paraffin Embedding

Tissue samples were fixed in 4% buffered formaldehyde for at least 72 h and then rinsed in running tap water for 24 h. The material was dehydrated in a graded ethanol series of 75%, 96%, and 100% using a vacuum tissue processor ETP RVG3 (Intelsint, Pomezia, Italy), cleared in xylene, and embedded in paraffin. Serial sections, 4 µm thick, were cut using a Slide 2003 sliding microtome (Pfm A.G., Cologne, Germany). The stained sections were examined using a Zeiss Axio Scope A1 light microscope (Carl Zeiss, Jena, Germany), and images were acquired with Axio Vision Release 4.8.2 SP2 software (Carl Zeiss MicroImaging GmbH, Jena, Germany). Anatomical and histological terminology followed the *Nomina Anatomica Avium* [1] and the *Nomina Histologica Veterinaria* [32], where applicable.

### 2.5. Histological and Histochemical Staining

Histological and histochemical staining was performed to characterize the general organization of the upper eyelid, lower eyelid, and nictitating membrane, with particular attention to the epithelium, connective tissue framework, muscular components, blood vessels, goblet cells, and conjunctiva-associated lymphoid tissue. The following stains were used: Mayer’s hematoxylin and eosin, Heidenhain’s Azan trichrome staining (Bio-Optica Milano S.p.A., Milan, Italy; cat. no. 04-001802), Mallory trichrome with aniline blue (Bio-Optica Milano S.p.A., Milan, Italy; cat. no. 04-020802), Masson–Goldner trichrome staining (Sigma-Aldrich/Merck KGaA, Darmstadt, Germany; cat. no. 1.00485), and Movat pentachrome, modified Russell–Movat method (BioGnost Ltd., Zagreb, Croatia; cat. no. MOV-100T) [33,34]. Mayer’s hematoxylin and eosin was used as the primary stain for general epithelial, stromal, vascular, muscular, and lymphoid assessment. Azan, Mallory trichrome, and Masson–Goldner trichrome were used to better distinguish collagenous connective tissue from muscle, blood vessels, and surrounding soft tissue. Modified Russell–Movat pentachrome was used to evaluate the connective tissue framework more broadly, particularly collagen, elastic fibers, ground substance, and vascular wall components. The use of several complementary stains was therefore intended to confirm the tissue composition of selected structures, especially the tarsal plate, eyelid stroma, marginal plait, and nictitating membrane folds, rather than to imply separate analytical categories between species. Representative figures were selected primarily for tissue preservation and clarity of the structure shown; H&E sections were used wherever possible, whereas trichrome or pentachrome sections were retained when they demonstrated connective tissue components more clearly. Briefly, in the trichrome stains, collagenous connective tissue stained blue with Azan and Mallory methods and green with Masson–Goldner staining, whereas muscle and cytoplasmic components stained red to pink and nuclei stained dark. In the modified Russell–Movat pentachrome method, collagen was visualized in yellow, elastic fibers and nuclei in dark to black tones, ground substance/mucosubstances in blue to green tones, and muscle in red. These color contrasts were used to distinguish the connective-tissue framework, vascular wall components, stromal organization, and fibrous elements of the eyelids and nictitating membrane.

### 2.6. Histological Evaluation of the Eyelids, Nictitating Membrane, and CALT

Histological evaluation of the upper and lower eyelids included the anterior and posterior palpebral surfaces, marginal zone, skin folds, feather follicles, musculofibrous layer, blood vessels, connective tissue framework, tarsal plate of the lower eyelid, goblet cells, and the presence and distribution of melanin pigment. The presence of adipose tissue, bundles of skeletal muscle, dense connective tissue, and vascular structures was also recorded. For the nictitating membrane, the following features were evaluated: morphology of the marginal plait and leading edge, palpebral surface, bulbar surface, palpebral folds, bulbar folds, epithelial organization, stromal characteristics, blood vessels, and the presence and distribution of melanin pigment. The assessment followed the criteria used in previous avian eyelid studies, with modifications appropriate for the present material [10]. The upper eyelid, lower eyelid, and nictitating membrane were also examined for the presence and organization of conjunctiva-associated lymphoid tissue (CALT). CALT was assessed separately in each anatomical region and described as diffuse lymphoid populations, lymphoid aggregates, or lymphoid nodules. Diffuse CALT was defined as scattered lymphoid cells within the conjunctival substantia propria. Lymphoid aggregates or nodules were defined as compact subepithelial accumulations of lymphoid cells located beneath the conjunctival epithelium. Because a typical follicular architecture with a clear germinal center was not consistently identifiable in the examined routine sections, the term lymphoid follicle was avoided for the present material unless referring to previously published descriptions. High endothelial venules (HEVs) were assessed in relation to lymphoid aggregates and nodules. HEVs were identified morphologically as small venules lined by plump, cuboidal to high endothelial cells and located within or close to organized lymphoid tissue. Because the study was based on routine histological methods, HEVs were interpreted based on their morphology and topographical relationship with lymphoid tissue [13,14]. Thus, in the present study, the term HEV refers to morphologically HEV-like venules and should not be understood as immunophenotypically or molecularly confirmed HEVs.

### 2.7. Microscopic Documentation and Data Analysis

Representative micrographs were selected to document the main histological features of each anatomical structure and species. All variables were assessed descriptively. Because the number of specimens differed among species, the results were interpreted as comparative descriptive observations rather than population-level differences. Caution was used when interpreting species represented by a single specimen: Black Woodpecker, Middle Spotted Woodpecker, and Gray-headed Woodpecker. The same caution was applied to Lesser Spotted Woodpecker, which was represented by two individuals; therefore, observations in species represented by one or two individuals were treated as specimen-level observations and not as definitive species-wide traits. Statistical comparisons among species were not performed because of the unequal sample sizes and the descriptive character of the study.

## 3. Results

**Figure 1:**
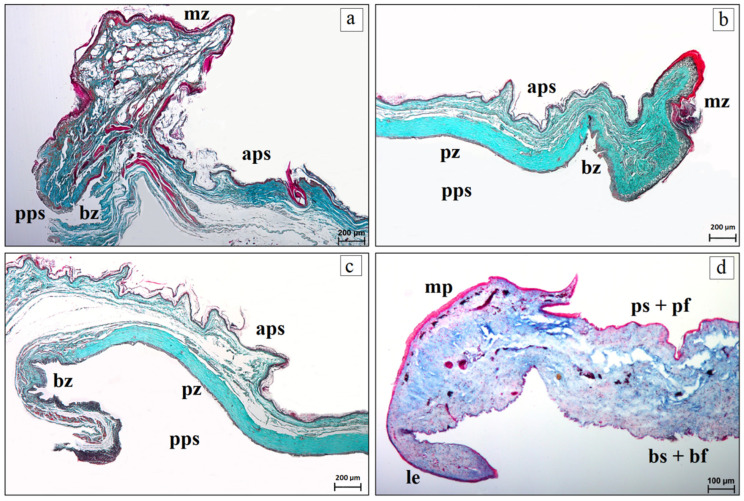
Representative histological organization of the upper eyelid, lower eyelid, and nictitating membrane in the six examined woodpecker species. (**a**) upper eyelid; (**b**,**c**) lower eyelid; (**d**) nictitating membrane. Abbreviations: aps—anterior palpebral surface; bs + bf—bulbar surface and bulbar folds; bz—bulbar zone; le—leading edge; mp—marginal plait; mz—marginal zone; pps—posterior palpebral surface; ps + pf—palpebral surface and palpebral folds; pz—plate zone. Staining: (**a**–**c**) Movat pentachrome (modified Russell–Movat); (**d**) Mallory trichrome with aniline blue. Scale bars: (**a**–**c**) 200 µm; (**d**) 100 µm. General color interpretation for special stains used in the figures: in Heidenhain’s Azan trichrome and Mallory trichrome with aniline blue, collagenous connective tissue appears blue, whereas muscle and cytoplasmic components are red to pink; in Masson–Goldner trichrome, collagenous connective tissue appears green and muscle/cytoplasmic components red to pink. In modified Russell–Movat pentachrome, collagen is yellow, elastic fibers and nuclei are dark to black, ground substance/mucosubstances are blue to green, and muscle is red. These color differences were used to document the eyelid stroma, tarsal plate, vascular walls, marginal plait, and folds of the nictitating membrane.

In all six examined woodpecker species, the upper and lower eyelids consisted of a skin surface, corresponding to the anterior palpebral surface, an eyelid margin, referred to as the marginal zone, and a conjunctival surface, corresponding to the posterior palpebral surface, which included the bulbar and plate zones. The nictitating membrane was composed of a leading edge, marginal plait, palpebral surface with palpebral folds, and bulbar surface with bulbar folds (Figure 1a–d). No clear histological differences related to sex or age were observed in the examined material. All differences among species are reported descriptively and should not be read as definitive species-level traits, particularly for species represented by one or two individuals.

### 3.1. Upper and Lower Eyelids

**Figure 2:**
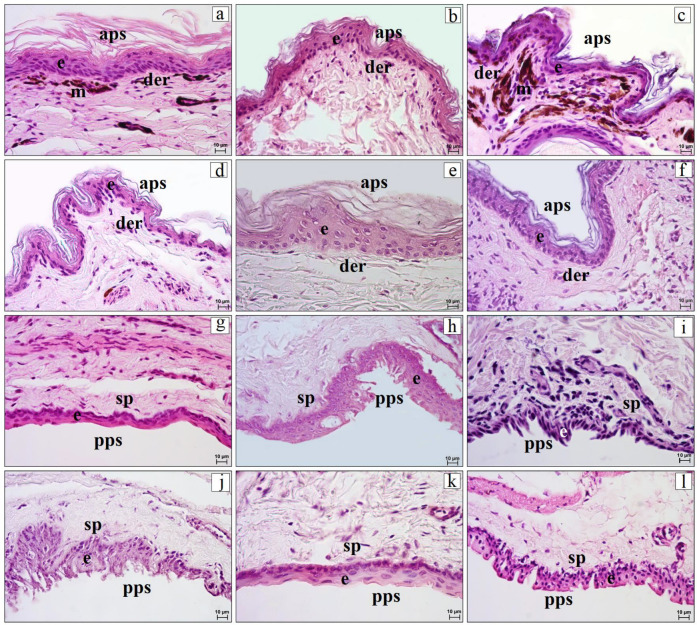
Histological features of the anterior and posterior palpebral surfaces of the eyelids in the examined woodpecker species. (**a**–**f**) anterior palpebral surface; (**g**–**l**) posterior palpebral surface. This figure is intended to compare the epithelial and connective-tissue organization of the palpebral surfaces among species; it does not present a separate upper-versus-lower eyelid comparison because both eyelids showed the same basic epithelial pattern at these surfaces. Panels: (**a**,**g**) Gray-headed Woodpecker *(Picus canus*); (**b**,**h**) Eurasian Green Woodpecker (*Picus viridis*); (**c**,**i**) Black Woodpecker (*Dryocopus martius*); (**d**,**j**) Great Spotted Woodpecker (*Dendrocopos major*); (**e**,**k**) Middle Spotted Woodpecker (*Dendrocoptes medius*); (**f**,**l**) Lesser Spotted Woodpecker (*Dryobates minor*). Abbreviations: aps—anterior palpebral surface; e—epithelium; der—dermis; m—melanin pigment; pps—posterior palpebral surface; sp—substantia propria. Staining: (**a**–**l**) Mayer’s hematoxylin and eosin. Scale bars: 10 µm.

**Figure 3:**
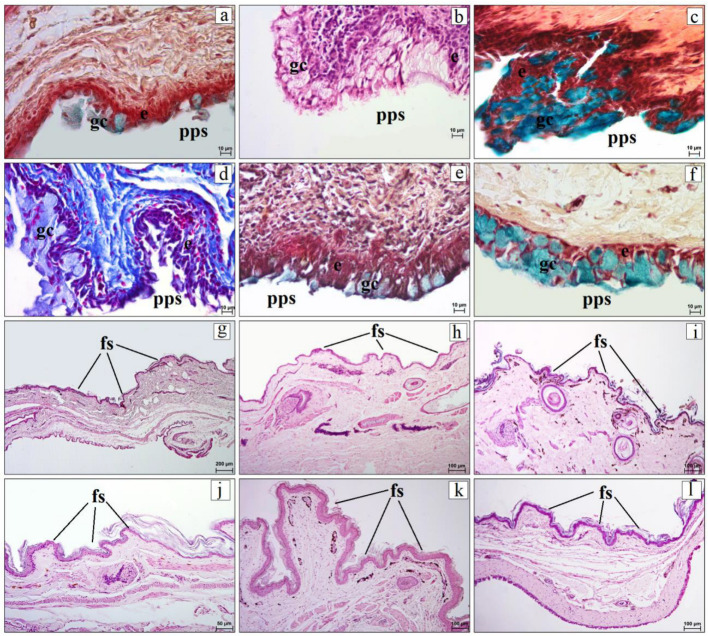
Goblet cells in the posterior palpebral surface and skin folds of the eyelids in the examined woodpecker species. (**a**–**f**) goblet cells within the epithelium of the posterior palpebral surface; (**g**–**l**) skin folds of the eyelids. Panels: (**a**,**g**) Gray-headed Woodpecker *(Picus canus*); (**b**,**h**) Eurasian Green Woodpecker (*Picus viridis*); (**c**,**i**) Black Woodpecker (*Dryocopus martius*); (**d**,**j**) Great Spotted Woodpecker (*Dendrocopos major*); (**e**,**k**) Middle Spotted Woodpecker (*Dendrocoptes medius*); (**f**,**l**) Lesser Spotted Woodpecker (*Dryobates minor*). Abbreviations: e—epithelium; fs—skin folds; gc—goblet cells; pps—posterior palpebral surface. Staining: (**a**,**c**,**e**,**f**) Movat pentachrome (modified Russell–Movat); (**b**,**g**–**l**) Mayer’s hematoxylin and eosin; (**d**) Mallory trichrome with aniline blue. Scale bars: (**a**–**f**) 10 µm; (**g**) 200 µm; (**h**,**i**,**k**,**l**) 100 µm; (**j**) 50 µm.

**Figure 4:**
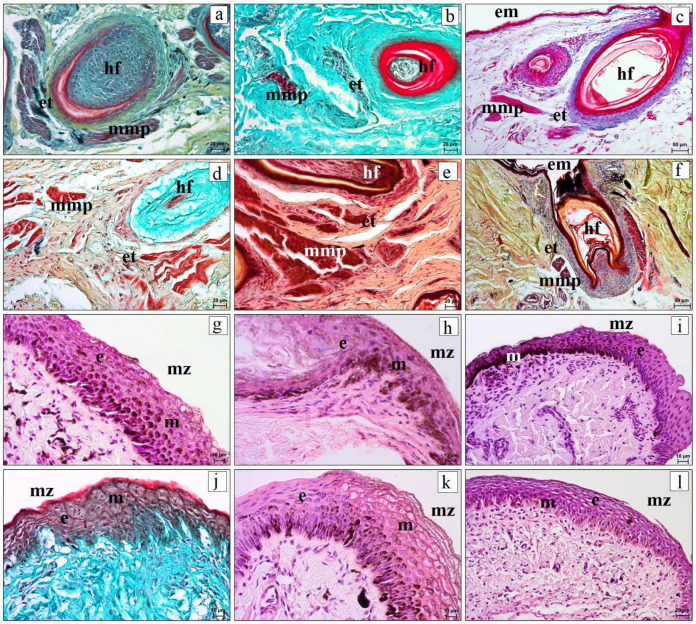
Histological features of hair-like feathers, the associated musculi pennarum and elastic tendon, and the marginal zone of the eyelids in the examined woodpecker species. (**a**–**f**) hair-like feathers located at the eyelid margin, on the anterior palpebral side of the marginal zone, with associated elastic tendon and musculi pennarum; (**g**–**l**) marginal zone epithelium with melanin pigment. Panels: (**a**,**g**) Gray-headed Woodpecker *(Picus canus*); (**b**,**h**) Eurasian Green Woodpecker (*Picus viridis*); (**c**,**i**) Black Woodpecker (*Dryocopus martius*); (**d**,**j**) Great Spotted Woodpecker (*Dendrocopos major*); (**e**,**k**) Middle Spotted Woodpecker (*Dendrocoptes medius*); (**f**,**l**) Lesser Spotted Woodpecker (*Dryobates minor*). Abbreviations: e—epithelium; em—eyelid margin; et—elastic tendon; hf—hair-like feathers; m—melanin pigment; mmp—musculi pennarum; mz—marginal zone. Staining: (**a**,**d**,**e**,**f**) Movat pentachrome (modified Russell–Movat); (**b**,**j**) Masson–Goldner trichrome; (**c**) Mallory trichrome with aniline blue; (**g**–**i**,**k**,**l**) Mayer’s hematoxylin and eosin. Scale bars: (**a**,**b**,**d**,**e**,**l**) 20 µm; (**c**,**f**) 50 µm; (**g**–**k**) 10 µm.

**Figure 5:**
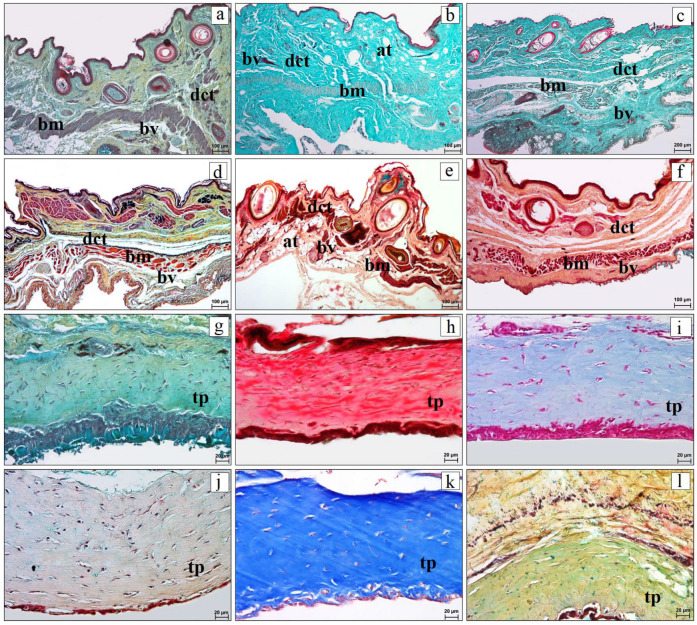
Histological organization of the connective, muscular, vascular, and adipose components of the upper eyelid and the tarsal plate of the lower eyelid in the examined woodpecker species. (**a**–**f**) connective tissue framework with bundles of muscle, blood vessels, and adipose tissue; (**g**–**l**) tarsal plate of the lower eyelid, composed of compact dense connective tissue. Panels: (**a**,**g**) Gray-headed Woodpecker (*Picus canus*); (**b**,**h**) Eurasian Green Woodpecker (*Picus viridis*); (**c**,**i**) Black Woodpecker (*Dryocopus martius*); (**d**,**j**) Great Spotted Woodpecker (*Dendrocopos major*); (**e**,**k**) Middle Spotted Woodpecker (*Dendrocoptes medius*); (**f**,**l**) Lesser Spotted Woodpecker (*Dryobates minor*). Abbreviations: at—adipose tissue; bm—bundles of muscle; bv—blood vessels; dct—dense connective tissue; tp—tarsal plate of the lower eyelid. Staining: (**a**,**d**–**h**,**j**,**l**) Movat pentachrome (modified Russell-Movat); (**b**,**c**) Masson–Goldner trichrome; (**i**,**k**) Mallory trichrome with aniline blue. Scale bars: (**a**,**b**,**d**–**f**) 100 µm; (**c**) 200 µm; (**g**–**l**) 20 µm.

**Figure 6:**
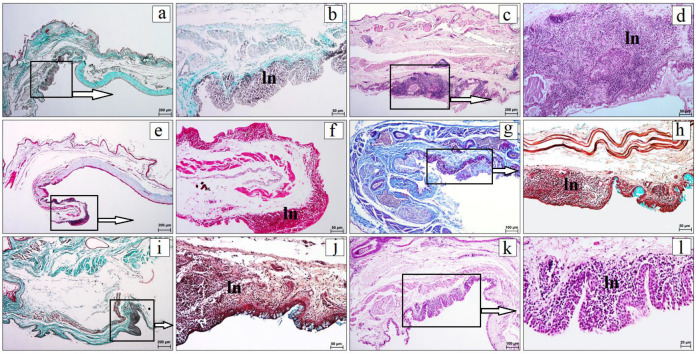
Lymphoid nodules in the lower eyelid of the examined woodpecker species. (**a**,**c**,**e**,**g**,**i**,**k**) general view of the lower eyelid with lymphoid regions indicated by boxes and arrows; (**b**,**d**,**f**,**h**,**j**,**l**) higher magnification of lymphoid nodules/aggregates. Panels: (**a**,**b**) Gray-headed Woodpecker (*Picus canus*); (**c**,**d**) Eurasian Green Woodpecker (*Picus viridis*); (**e**,**f**) Black Woodpecker (*Dryocopus martius*); (**g**,**h**) Great Spotted Woodpecker (*Dendrocopos major*); (**i**,**j**) Middle Spotted Woodpecker (*Dendrocoptes medius*); (**k**,**l**) Lesser Spotted Woodpecker (*Dryobates minor*). Abbreviations: ln—lymphoid nodule. Staining: (**a**,**b**,**i**) Masson–Goldner trichrome; (**c**,**d**,**k**,**l**) Mayer’s hematoxylin and eosin; (**e**–**g**) Mallory trichrome with aniline blue; (**h**,**j**) Movat pentachrome (modified Russell–Movat). Scale bars: (**a**,**c**,**e**,**i**) 200 µm; (**g**,**k**) 100 µm; (**b**,**d**,**f**,**h**,**j**) 50 µm; (**l**) 20 µm.

**Figure 7:**
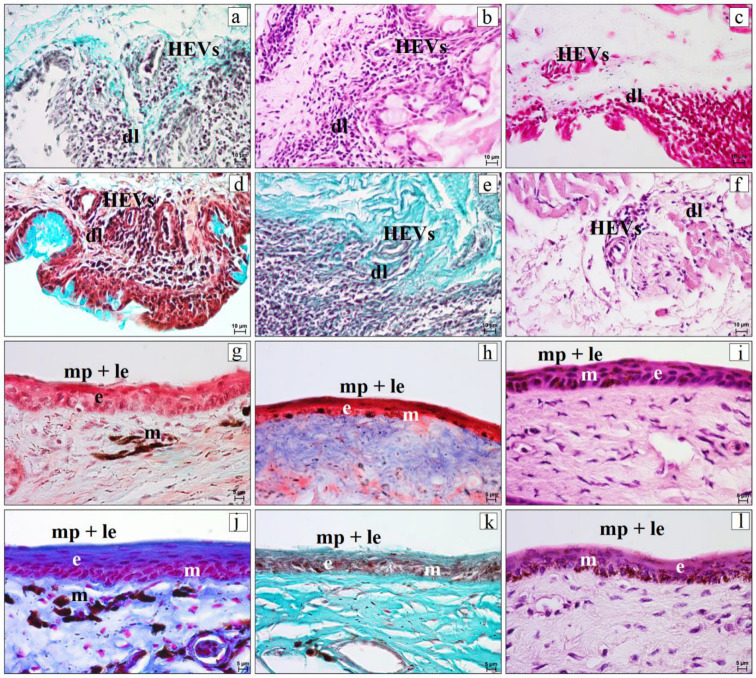
Diffuse lymphocytes and morphologically identified HEV-like venules in the lower eyelid, and histological features of the leading edge and marginal plait of the nictitating membrane in the examined woodpecker species. (**a**–**f**) Diffuse lymphocytes and HEV-like venules in the lower eyelid; (**g**–**l**) epithelium and melanin pigmentation in the marginal plait and leading edge of the nictitating membrane. Panels: (**a**,**g**) Gray-headed Woodpecker (*Picus canus*); (**b**,**h**) Eurasian Green Woodpecker (*Picus viridis*); (**c**,**i**) Black Woodpecker (*Dryocopus martius*); (**d**,**j**) Great Spotted Woodpecker (*Dendrocopos major*); (**e**,**k**) Middle Spotted Woodpecker (*Dendrocoptes medius*); (**f**,**l**) Lesser Spotted Woodpecker (*Dryobates minor*). Abbreviations: dl—diffuse lymphocytes; e—epithelium; HEVs—morphologically identified high endothelial venule-like vessels; m—melanin pigment; mp + le—marginal plait and leading edge. Staining: (**a**,**e**,**k**) Masson–Goldner trichrome; (**b**,**f**,**i**,**l**) Mayer’s hematoxylin and eosin; (**c**,**j**) Mallory trichrome with aniline blue; (**d**,**g**,**h**) Movat pentachrome (modified Russell–Movat). Scale bars: (**a**–**f**) 10 µm; (**g**–**l**) 5 µm.

In all examined species, the skin surface of both the upper and lower eyelids was covered by keratinized stratified squamous epithelium (Figure 2a–f). In the examined material, the greatest number of epithelial cell layers forming the anterior palpebral epithelium was observed in the single available *P. canus* specimen (9–10 layers). In *P. viridis*, *D. martius*, and *D. medius*, this epithelium consisted of 6–8 layers, whereas the lowest number of layers was recorded in *D. major* and *D. minor* (5–6 layers) (Figure 2a–f). Directly beneath the external epithelium, the dermis was present and contained collagen, reticular, and elastic fibers, as well as histiocytes and fibrocytes. In *P. canus* and *D. martius*, heavily pigmented cells containing dense cytoplasmic melanin were observed within the dermis (Figure 2a,c). The conjunctival surface of both eyelids was lined by non-keratinized stratified squamous epithelium containing goblet cells (Figure 2g–l and Figure 3a–f). The connective tissue underlying the conjunctival epithelium was referred to as the conjunctival substantia propria. Goblet cells were observed as single cells or small groups within the epithelium of the posterior palpebral surface. In *P. canus*, *D. martius*, *D. medius*, and *D. minor*, the number of nucleated epithelial cell layers ranged from 5 to 6. In *P. viridis* and *D. major*, it ranged from 8 to 11 layers (Figure 2g–l). The upper and lower eyelids were also characterized by the presence of skin folds (Figure 3g–l). Short and narrow skin folds were most commonly observed in *P. canus*, *P. viridis*, and *D. martius* (Figure 3g–i), whereas longer and wider skin folds occurred in *D. major*, *D. medius*, and *D. minor* (Figure 3j–l). Tarsal glands were not observed in the eyelid margins of any of the examined species. The eyelid margin was sparsely feathered and was lined by hair-like feathers associated with the musculi pennarum and elastic tendon (Figure 4a–f). These hair-like feathers were located at the eyelid margin, in the transition between the anterior palpebral skin and the marginal zone, rather than on the conjunctival surface. The marginal zone was covered by stratified squamous epithelium with superficial flattening and focal keratinization/parakeratinization in some sections (Figure 4g–l). In *P. canus*, *P. viridis*, *D. martius*, *D. medius*, and *D. minor*, the epithelium of the marginal zone consisted of 13–19 layers of nucleated cells, whereas in *D. major*, it consisted of 9–11 layers (Figure 4g–l). Melanin pigment was observed in the marginal zone epithelium of most examined species and was generally more evident in this region than in the anterior palpebral surface (Figure 4g–l). Its distribution varied among species. In *P. canus*, *P. viridis*, *D. major*, and *D. medius*, heavily pigmented cells containing dense cytoplasmic melanin were usually distributed throughout all epithelial layers. In *D. martius*, they were particularly prominent in the basal epithelial layers, whereas in *D. minor*, they were scattered mainly among the basal cells of the marginal zone epithelium (Figure 4g–l). The stroma of the upper and lower eyelids was composed mainly of irregular dense collagenous connective tissue with irregularly arranged elastic and reticular fibers, bundles of skeletal muscle, nerves, and blood vessels (Figure 5a–f). The muscle bundles were interpreted topographically as parts of the levator palpebrae dorsalis, depressor palpebrae ventralis, and orbicularis oculi muscles. In *P. viridis* and *D. medius*, focal accumulations of adipose tissue were also observed (Figure 5b,e). The tarsal plate was present only in the lower eyelid. In transverse histological sections, it was oval to elongated and was composed of compact regular dense collagenous connective tissue with delicate elastic and reticular fibers, fibrocytes, and blood vessels (Figure 5g–l). In Figure 5, the term dense connective tissue refers to the tissue composition of the tarsal plate rather than to a separate anatomical structure. The tarsal plate was more compact than the surrounding eyelid stroma. In the lower eyelids of the examined species, CALT was found in close relation to the conjunctival surface. It was represented by subepithelial lymphoid nodules/aggregates and diffuse lymphocytes. The lymphoid nodules were oval to irregular in shape and were located beneath the posterior palpebral epithelium. Because a typical follicular arrangement with a clearly visible germinal center was not consistently observed in the routine sections, these structures are described as lymphoid nodules/aggregates rather than lymphoid follicles. Venules with HEV-like morphology were observed within or close to lymphoid tissue (Figure 6a–l and Figure 7a–f).

### 3.2. Nictitating Membrane

**Figure 8:**
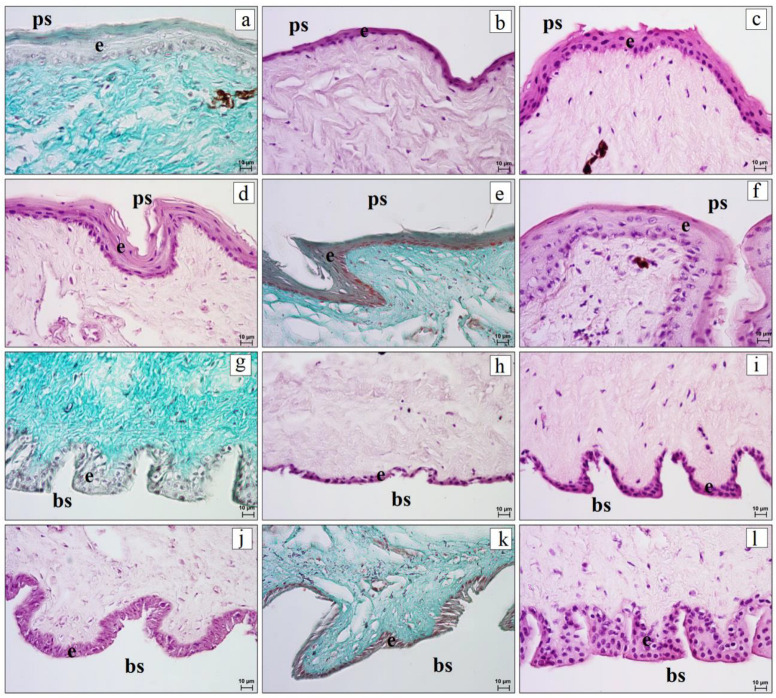
Histological features of the palpebral and bulbar surfaces of the nictitating membrane in the examined woodpecker species. (**a**–**f**) Palpebral surface of the nictitating membrane; (**g**–**l**) bulbar surface of the nictitating membrane. Panels: (**a**,**g**) Gray-headed Woodpecker (*Picus canus*); (**b**,**h**) Eurasian Green Woodpecker (*Picus viridis*); (**c**,**i**) Black Woodpecker (*Dryocopus martius*); (**d**,**j**) Great Spotted Woodpecker (*Dendrocopos major*); (**e**,**k**) Middle Spotted Woodpecker (*Dendrocoptes medius*); (**f**,**l**) Lesser Spotted Woodpecker (*Dryobates minor*). Abbreviations: bs—bulbar surface; e—epithelium; ps—palpebral surface. Staining: (**a**,**e**,**g**,**k**) Masson–Goldner trichrome; (**b**–**d**,**f**,**h**–**j**,**l**) Mayer’s hematoxylin and eosin. Scale bars: 10 µm.

**Figure 9:**
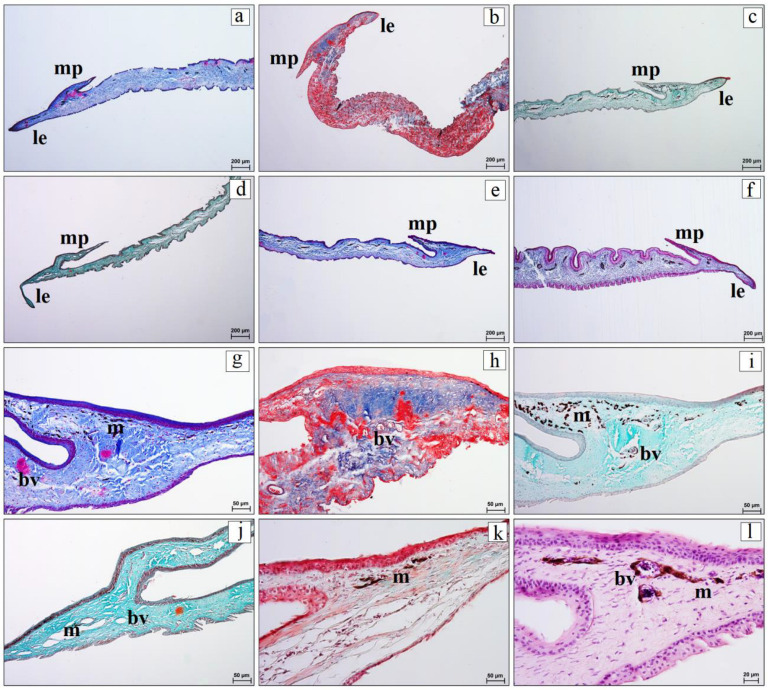
Histological organization of the nictitating membrane in the examined woodpecker species. (**a**–**f**) General view of the nictitating membrane showing the leading edge and marginal plait; (**g**–**l**) higher magnification of the nictitating membrane showing blood vessels and melanin pigment. Panels: (**a**,**g**) Gray-headed Woodpecker (*Picus canus*); (**b**,**h**) Eurasian Green Woodpecker (*Picus viridis*); (**c**,**i**) Black Woodpecker (*Dryocopus martius*); (**d**,**j**) Great Spotted Woodpecker (*Dendrocopos major*); (**e**,**k**) Middle Spotted Woodpecker (*Dendrocoptes medius*); (**f**,**l**) Lesser Spotted Woodpecker (*Dryobates minor*). Abbreviations: bv—blood vessels; le—leading edge; m—melanin pigment; mp—marginal plait. Staining: (**a**,**b**,**e**–**h**) Mallory trichrome with aniline blue; (**c**,**d**,**i**,**j**) Masson–Goldner trichrome; (**k**) Movat pentachrome (modified Russell–Movat); (**l**) Mayer’s hematoxylin and eosin. Scale bars: (**a**–**f**) 200 µm; (**g**–**k**) 50 µm; (**l**) 20 µm.

**Figure 10:**
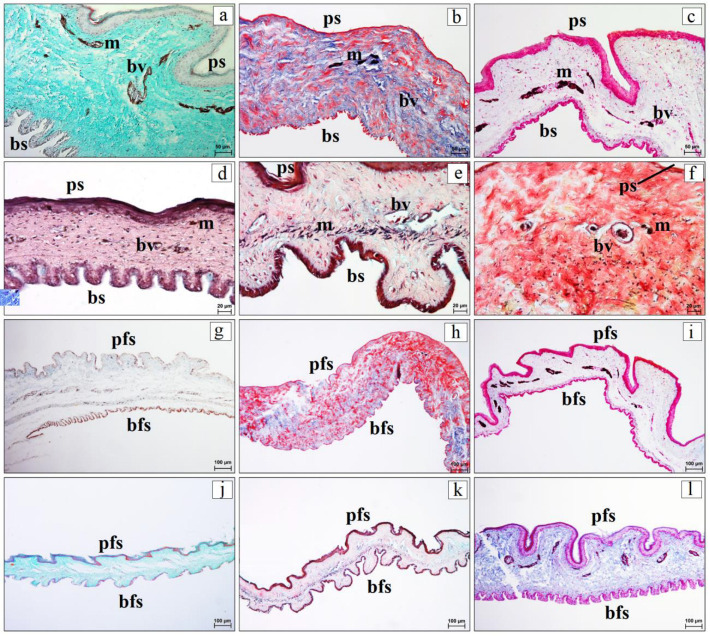
Histological features of the palpebral and bulbar surfaces and folds of the nictitating membrane in the examined woodpecker species. (**a**–**f**) palpebral and bulbar surfaces of the nictitating membrane with visible blood vessels and melanin pigment; (**g**–**l**) palpebral and bulbar folds of the nictitating membrane. Panels: (**a**,**g**) Gray-headed Woodpecker (*Picus canus*); (**b**,**h**) Eurasian Green Woodpecker (*Picus viridis*); (**c**,**i**) Black Woodpecker (*Dryocopus martius*); (**d**,**j**) Great Spotted Woodpecker (*Dendrocopos major*); (**e**,**k**) Middle Spotted Woodpecker (*Dendrocoptes medius*); (**f**,**l**) Lesser Spotted Woodpecker (*Dryobates minor*). Abbreviations: bfs—bulbar folds; bs—bulbar surface; bv—blood vessels; m—melanin pigment; pfs—palpebral folds; ps—palpebral surface. Staining: (**a**,**g**,**j**) Masson–Goldner trichrome; (**c**,**i**,**l**) Mallory trichrome with aniline blue; (**b**,**d**,**e**,**f**,**k**) Movat pentachrome (modified Russell–Movat). Scale bars: (**a**–**c**) 50 µm; (**d**–**f**) 20 µm; (**g**–**l**) 100 µm.

The marginal plait and leading edge of the nictitating membrane were lined by non-keratinized stratified squamous epithelium (Figure 7g–l). The number of nucleated epithelial cell layers in this region varied among species. In *P. canus*, *D. martius*, *D. medius*, and *D. minor*, the epithelium consisted of 5–9 layers, whereas in *P. viridis* it consisted of 4–7 layers. The highest number of epithelial layers was observed in *D. major*, where the epithelium reached 10–11 layers (Figure 7g–l). In *P. viridis*, *D. martius*, *D. major*, *D. medius*, and *D. minor*, weak melanin pigmentation was observed in the epithelium of the marginal plait and leading edge (Figure 7g–l). The palpebral surface of the nictitating membrane was lined by non-keratinized stratified squamous epithelium, whereas the bulbar surface was lined by non-keratinized stratified columnar epithelium (Figure 8a–l). The number of epithelial cell layers on the palpebral surface ranged from 3–4 in *P. viridis* to 8–11 in the remaining examined woodpecker species. On the bulbar surface, the epithelium consisted of 2–3 layers in *P. viridis*, *D. martius*, and *D. medius*, and 5–8 layers in *P. canus*, *D. major*, and *D. minor* (Figure 8a–l). Surface microvilli on the bulbar side of the nictitating membrane were not assessable in the present material because they are ultrastructural features beyond the reliable resolution of routine light microscopy and because *post-mortem* preservation varied among specimens. In the single examined *D. martius* individual, the marginal plait and leading edge of the nictitating membrane were thicker than in the remaining examined specimens (Figure 9a–f). The stroma of the marginal plait was composed of regular dense collagenous connective tissue with reticular and elastic fibers, fibrocytes, and blood vessels (Figure 9g–l). In *P. canus*, *D. martius*, *D. major*, *D. medius*, and *D. minor*, accumulations of melanin pigment were observed in the stroma of the marginal plait (Figure 9g,i–l). The stroma of the nictitating membrane was composed of dense connective tissue with dominant collagen fibers, clearly visible elastic and reticular fibers, fibrocytes, and blood vessels. In several species, heavily pigmented cells were located mainly around blood vessels (Figure 10a–f). The palpebral surface formed relatively thick folds, whereas the bulbar surface formed thinner and more numerous folds (Figure 10g–l). The number of palpebral folds varied from 8–9 in *D. minor* to 12–18 in the remaining examined woodpecker species. The number of bulbar folds was higher and ranged from 26 to 38 (Figure 10g–l). No organized lymphoid nodules/aggregates were observed in the nictitating membrane in the examined material.

## 4. Discussion

In all examined European woodpecker species, the general organization of the eyelids corresponded to the basic avian pattern described by Baumel et al. [1], Jochems and Phillips [2], Hall et al. [4], Bacha & Bacha [35], Gültiken et al. [36], and Bayón et al. [37]. However, some differences were observed between the examined species, mainly in epithelial thickness, development of skin folds, distribution of melanin pigment, organization of the marginal zone, and structure of the nictitating membrane. Because the material was unevenly distributed among species, all interspecific interpretations are made cautiously, and observations in species represented by one or two individuals are not treated as definitive species-level traits.

Compared with other birds examined in previous studies, including birds of prey and selected ornamental and wild species, the woodpeckers shared the basic avian eyelid plan but showed a distinctive combination of features: a keratinized anterior palpebral surface, a sparsely feathered eyelid margin with hair-like feathers, a compact tarsal plate in the lower eyelid, lower-eyelid CALT, and a folded nictitating membrane [7,10]. In birds of prey, the eyelids and nictitating membrane have been interpreted mainly in relation to visual protection during hunting, flight, and exposure to airborne particles, whereas in ornamental, aquatic, terrestrial, and arboreal birds, differences in eyelid folds, pigmentation, goblet cells, and CALT organization have been discussed in relation to habitat and ocular surface exposure [7,10]. In woodpeckers, the same general protective components are present, but their functional context may be different because the periocular region is repeatedly exposed to bark fragments, wood dust, substrate particles, and mechanical vibration during climbing, drilling, and pecking. Thus, the nictitating membrane of woodpeckers may differ from that of other bird groups mainly in the relative development of folds, marginal plait morphology, and stromal support, which could facilitate precorneal tear film spreading and removal of particulate material while maintaining a smooth ocular surface during rapid head movements. These interpretations remain comparative and descriptive because the present study did not include functional measurements.

The eyelids consisted of an external skin surface, a middle musculofibrous layer, and an internal conjunctival surface. The anterior palpebral surface of both eyelids was covered by keratinized stratified squamous epithelium. This type of epithelium is typical for the external surface of the eyelid, which is continuous with the skin and is exposed to environmental factors. A similar organization of the eyelid surface has been described in other avian species by Klećkowska-Nawrot et al. [7,10]. The observed differences in the number of epithelial cell layers may reflect a combination of ecological exposure, body size, and phylogenetic background among the studied species. The higher number of epithelial layers in the single examined *P. canus* specimen may be related to the more terrestrial, myrmecophagous habits described for this species [32], because foraging on the ground and excavating anthills may expose the periocular surface to soil, sand, and chemical irritants such as formic acid. In contrast, the lower number of epithelial layers recorded in *D. major* and *D. minor* should not be interpreted as evidence of reduced protection; rather, it may represent a different balance between epithelial thickness, tissue flexibility, and the mechanical demands associated with frequent pecking and drilling [38,39]. Because no biomechanical testing was performed, this interpretation remains hypothetical. The posterior palpebral surface was lined with a non-keratinized stratified squamous epithelium containing goblet cells. This is consistent with the role of the palpebral conjunctiva in maintaining the ocular surface and contributing to the mucous component of the precorneal tear film, as described by Bayón et al. [37], Jochems and Phillips [2], and Klećkowska-Nawrot et al. [7,10]. While these goblet cells were distinct on the conjunctival surface of the eyelids, their presence in the nictitating membrane was inconsistent across the analyzed sections. This variation is likely related to the delicate structure of the membrane and inconsistent preservation in *post-mortem* material. Consequently, these findings should not be interpreted as an absolute absence of goblet cells in the nictitating membrane.

Skin folds were present in the eyelids of all examined species, although their morphology varied considerably. Shorter and narrower folds occurred in *P. canus*, *P. viridis*, and *D. martius*, whereas longer and wider folds were observed in *D. major*, *D. medius*, and *D. minor.* Comparable interspecific variation in eyelid surface microstructure has also been reported in other avian groups [7,10]. The observed pattern only partly corresponds with ecological differences among European woodpeckers. Species of the genus *Picus* [26,27] rely more heavily on ground foraging and myrmecophagy, whereas *Dendrocopos*, *Dendrocoptes*, and *Dryobates* are more strongly associated with arboreal substrates [28,29,30]. However, the relationship between fold morphology and eyelid mobility cannot be inferred directly from histological sections. Therefore, the shorter and narrower folds observed in *P. canus* and *P. viridis* should be interpreted only as a morphological observation, not as direct evidence of greater eyelid mobility or more rapid visual monitoring. *D. martius* also exhibited relatively short and narrow folds despite being among the most specialized excavators of European woodpeckers [31], which further indicates that eyelid morphology cannot be explained solely by foraging substrate or excavation intensity. The longer and wider folds observed in *D. major*, *D. medius*, and *D. minor* may increase the epithelial and conjunctival surface available for local mechanical protection and precorneal tear film distribution, but this interpretation remains hypothetical. Woodpeckers have been reported to close their eyelids immediately before impact [39], and more extensive folds could help shield the ocular region from wood fragments, bark particles, and other debris generated during excavation. Taken together, the observed variation may reflect the combined influence of ecological specialization, body size, and phylogenetic history rather than any single ecological factor, but this remains a hypothesis requiring confirmation in larger and more balanced samples.

The eyelid margin was sparsely feathered and contained hair-like feathers associated with the *musculi pennarum* and an elastic tendon. Similar modified feathers or feather-like structures in the eyelid region have previously been described in birds by Bayón et al. [37] and Klećkowska-Nawrot et al. [7]. These hair-like feathers may serve a function analogous to that of eyelashes in mammals. Their association with the *musculi pennarum* and elastic tendon may indicate that they are not merely passive structures, although their mobility was not tested in the present study. In woodpeckers, such mobility could enhance protection of the periocular region by helping to prevent bark fragments, wood dust, and other debris generated during foraging and drilling activities from reaching the ocular surface.

The marginal zone was covered by stratified squamous epithelium with superficial flattening and, in some sections, focal keratinization or parakeratinization. This interpretation is more consistent with the microscopic appearance of the examined sections than the previously used term stratified columnar epithelium. The marginal zone also exhibited distinct melanin pigmentation. Cells containing dense cytoplasmic melanin were prominent throughout this epithelial region, although their distribution varied among the examined species. Similar pigmentation of the eyelids and conjunctiva has previously been reported in other avian taxa [7,10]. In the present study, melanin was additionally observed within the dermis of the anterior palpebral surface in certain species and in the stroma of the nictitating membrane. The greater abundance of melanin in the marginal zone compared with the anterior palpebral surface may indicate a protective role against environmental stressors, including ultraviolet radiation and oxidative damage. Although the functional significance of the observed interspecific variation remains uncertain, differences in melanin distribution may reflect variation among the examined specimens, possible species-related tendencies, or phylogenetic background; they should not be interpreted as demonstrated adaptations. The stroma of the upper and lower eyelids consisted primarily of dense collagenous connective tissue interspersed with elastic and reticular fibers, skeletal muscle bundles, nerves, and blood vessels.

The lower eyelid contained a distinct fibrous tarsal plate, serving as a compact supportive element. This finding aligns with the general avian anatomy described by Baumel et al. [1], Bayón et al. [37], Hall et al. [4], and Gültiken et al. [36]. Consequently, the tarsal plate should not be viewed as a specialized adaptation unique to woodpeckers, but rather as a component of the avian lower eyelid that was clearly discernible in the examined species. In broader comparative anatomy, the tarsal plate or tarsus of non-avian species, especially mammals, is regarded as a dense connective tissue support that helps maintain eyelid shape, stabilizes the eyelid margin during blinking, and provides a structural framework associated with the palpebral conjunctiva and, in many mammals, tarsal glands [35]. In the examined woodpeckers, tarsal glands were not observed; therefore, the tarsal plate is best interpreted as a mechanical and supportive component of the lower eyelid rather than a gland-bearing structure. Although CALT has been reported in several avian species, its localization and organization in Picidae remain poorly documented. Therefore, a relevant contribution of the present study is the description of CALT in the lower eyelid of European woodpeckers. In the present material, CALT was characterized by lymphoid nodules/aggregates and diffuse lymphocytes, with morphologically identified HEV-like venules situated within or adjacent to lymphoid tissue. Because germinal centers were not consistently identifiable, the safer descriptive terms lymphoid nodules or aggregates are used instead of lymphoid follicles. Beyond its role in lubrication and maintaining the ocular surface, the palpebral conjunctiva serves as a component of the local immune barrier. Previous research has established CALT as an important element of mucosal immune protection across various taxa [40,41,42,43,44,45,46,47]. Within avian groups, a similar lower eyelid localization of organized lymphoid tissue was documented in chickens by Fix and Arp [14,15], while van Ginkel et al. [13] emphasized its broader role in avian ocular mucosal immunity. Furthermore, variations in lymphoid nodules/aggregates, diffuse lymphocytes, and HEV-like venules described by Klećkowska-Nawrot et al. [7,10] suggest that this tissue exhibits interspecific diversity linked to the biology and environmental exposure of different bird groups. Our results indicate that the lower eyelid of woodpeckers may represent an important site of local ocular immune defense. Together with the Harderian gland, CALT may constitute a component of ocular mucosal immunity in woodpeckers. The repeated exposure of the ocular surface to wood particles, bark fragments, and environmental microorganisms makes this immune barrier particularly relevant in Picidae. Notably, HEVs were identified herein solely based on their morphology and anatomical position. While this approach is useful for routine histological evaluation, its limitations should be acknowledged, as the endothelial phenotype of these vessels was not validated by immunohistochemistry. Consequently, the presence of HEVs in woodpecker CALT remains a morphological observation. Further investigations using specific endothelial and lymphoid markers are required to confirm their functional identity and fully characterize the organization of CALT in Picidae.

The non-keratinized stratified epithelium of the nictitating membrane may reflect a functional compromise between protection and surface smoothness. Its multilayered structure may enhance resistance to abrasion, while the lack of keratinization may help preserve a smooth surface and minimize interference with vision. Such an organization is consistent with the dual role of the avian nictitating membrane in protecting the ocular surface while maintaining visual function. The nictitating membrane in the examined woodpeckers featured a distinct marginal plait, a leading edge, and both palpebral and bulbar surfaces covered with folds. This general organization aligns with the established structure and function of the avian nictitating membrane, which protects the cornea and distributes the precorneal tear film [1,2,9,37,48,49]. Compared with previous observations in birds of prey and other wild or ornamental birds, the woodpecker nictitating membrane showed the same basic avian plan, but the numerous bulbar folds and the clear marginal plait may be especially relevant for the removal of fine particulate material generated during pecking, drilling, and bark-foraging [7,10]. In the present material, the epithelial organization differed between the two surfaces, with bulbar folds being more numerous than palpebral ones. This structural asymmetry may increase the surface area of the membrane and could facilitate debris removal and precorneal tear film distribution across the ocular surface. A similar protective role of the nictitating membrane in ocular surface maintenance was reported by Mahmoud et al. [49] in the Little Owl (*Athene noctua*). Microvilli on the bulbar surface have been described as a common epithelial specialization in avian conjunctival or nictitating membrane surfaces in ultrastructural studies, but such structures could not be evaluated reliably here because the present study was based on routine light microscopy and variably preserved *post-mortem* material. Therefore, no conclusion is drawn regarding the presence or absence of microvilli in the woodpecker nictitating membrane. Despite the overall similarity in nictitating membrane organization, some interspecific variation was evident. Among the examined specimens, the marginal plait and leading edge were thickest in the single *D. martius* individual. This observation is noteworthy given that the Black Woodpecker is distinguished by its ability to excavate deep cavities and forage extensively within tree trunks [31]. However, because only a single individual of this species was examined, the biological significance of this pattern remains uncertain. A similarly cautious interpretation is warranted for the other species represented by one or two individuals. Moreover, because the nictitating membrane is a delicate structure susceptible to *post-mortem* and processing-related artifacts, its preservation status varied among specimens. Consequently, the presence and histological organization of CALT within this structure could not be reliably assessed in all examined birds.

In conclusion, our findings indicate that the ocular adnexa of woodpeckers possess several structural characteristics potentially associated with ocular surface protection. Key among these are the keratinized anterior palpebral surface, a folded conjunctival surface, a pigmented marginal zone, the fibrous tarsal support of the lower eyelid, and the presence of CALT within the lower eyelid. Additionally, the nictitating membrane, through its distinct marginal plait, leading edge, and numerous bulbar folds, may further contribute to mechanical defense and precorneal tear film distribution. However, given that the tissue was obtained *post-mortem* from wild birds and sample sizes varied among species, these results should be considered baseline comparative and descriptive observations. Future studies utilizing larger cohorts, immunohistochemistry, and electron microscopy are warranted to definitively confirm the cellular composition of CALT, validate the identity of morphologically identified HEV-like venules, and elucidate the ultrastructure of the nictitating membrane.

## 5. Conclusions

This study provides comparative histological data on the upper eyelid, lower eyelid, and nictitating membrane in six European woodpecker species. It establishes the first comparative microscopic baseline for these ocular adnexal structures in European Picidae and places their organization in the possible biological context of ocular surface protection during climbing, drilling, pecking, and exposure to particulate material. In all examined species, the eyelids showed a similar basic organization, but species-related differences were observed in epithelial thickness, development of skin folds, distribution of melanin pigment, organization of the marginal zone, and structure of the nictitating membrane. The lower eyelid contained a distinct fibrous tarsal plate, which formed a compact supportive element within the eyelid. A relevant contribution of this study was the documentation of CALT in the lower eyelid of European woodpeckers. It was represented by lymphoid nodules/aggregates and diffuse lymphocytes, with morphologically identified HEV-like venules located within or close to lymphoid tissue. This suggests that the lower eyelid may be an important site of local ocular immune surveillance in woodpeckers. The nictitating membrane showed a distinct marginal plait and leading edge, regional differences in epithelial organization, stromal pigmentation, and numerous bulbar folds. These features may support precorneal tear film distribution, removal of small particles, and mechanical protection of the ocular surface. Together, the keratinized anterior palpebral surface, folded conjunctival surface, pigmented marginal zone, fibrous tarsal support, lower-eyelid CALT, and folded nictitating membrane suggest that the ocular adnexa of woodpeckers may form a coordinated structural and immune barrier rather than a set of isolated anatomical features. Because the material was obtained *post-mortem* from wild birds and the number of specimens differed among species, the results should be interpreted as comparative descriptive observations rather than population-level differences. In addition, the delicate structure and variable preservation of the nictitating membrane limited the reliable assessment of lymphoid structures in this region, and microvilli on the bulbar surface could not be assessed by routine light microscopy. Nevertheless, the present study provides baseline data for Picidae and expands the knowledge of avian ocular adnexa, especially in relation to local structural and immune protection of the ocular surface. These findings may serve as a reference for future comparative, functional, ultrastructural, and immunological studies of the eye and ocular adnexa in wild birds.

## 6. Limitations

The present study has several limitations that should be considered when interpreting the results. First, the material was obtained exclusively from birds found dead in the field. Therefore, the *post-mortem* interval differed among specimens and could not always be precisely determined. Although tissue preservation was assessed during dissection and preliminary histological screening, variable *post-mortem* changes may have affected some epithelial, vascular, and lymphoid structures. This limitation was particularly important for the nictitating membrane, which is a delicate structure and was not equally well preserved in all specimens. Second, the number of specimens differed markedly among species. The Great Spotted Woodpecker and European Green Woodpecker were represented by relatively larger samples, whereas the Lesser Spotted Woodpecker was represented by two individuals, and the Black Woodpecker, Middle Spotted Woodpecker, and Gray-headed Woodpecker by single specimens. Therefore, the results should be interpreted as comparative descriptive observations rather than population-level or species-wide conclusions. For the same reason, formal statistical comparisons among species were not performed. Third, although sampling was standardized to include the relevant anatomical components of the eyelids and nictitating membrane, strict standardization of the exact medial, central, or lateral eyelid region was not always possible because the specimens were collected *post-mortem* and differed in preservation. Direct measurements of eye size, palpebral fissure length, and head size were also not available in a standardized form and therefore could not be included in the analysis. Fourth, the study was based on routine histological methods. CALT and HEV-like venules were identified on the basis of their morphology and topographical relationship with lymphoid tissue. Therefore, the cellular composition of CALT and the endothelial phenotype of these HEV-like venules could not be confirmed by immunohistochemical or molecular methods. In addition, epithelial microvilli on the bulbar side of the nictitating membrane could not be evaluated using routine light microscopy. Finally, because the material was collected *post-mortem*, no *ante-mortem* ophthalmic examination or functional assessment of the eyelids and nictitating membrane was available. Thus, the relationship between the observed histological features and ocular surface function should be interpreted cautiously.

## 7. Future Recommendations

Future studies should include larger and more balanced sample sizes for each woodpecker species, ideally with standardized information on age, sex, *post-mortem* interval, fixation time, eye size, palpebral fissure length, and head size. Such material would allow more robust quantitative comparisons of epithelial thickness, conjunctival folding, melanin pigmentation, and the degree of CALT organization. Further investigations using immunohistochemical methods would be valuable to characterize the cellular composition of CALT, including T and B lymphocytes, plasma cells, macrophages, and other immune cells. Additional endothelial markers could also help confirm the identity and distribution of morphologically identified HEV-like venules within organized lymphoid tissue. Scanning or transmission electron microscopy could provide more detailed information on the surface morphology of the conjunctival epithelium, the marginal plait of the nictitating membrane, and epithelial specializations, including microvilli, involved in precorneal tear film distribution and removal of small particles from the ocular surface. In addition, future comparative studies including other Piciformes or broader avian groups may help determine whether the features observed in woodpeckers are characteristic of Picidae or represent more general patterns in birds exposed to similar ocular surface demands.

## Figures and Tables

**Table 1:** The table presents information on the sample sizes of individual species, the ages of the examined individuals, and their molecular sex as determined according to Klećkowska et al. [23]. The table additionally includes the gel lane number assigned to each examined individual. Species belonging to the family Picidae are listed in taxonomic order following AviList [25].

Genus	Species	Individual No.	Age	Sex	Lane in Figure S1
*Picus*	*Picus canus*	1	adult	female	36
*Picus*	*Picus viridis*	1	adult	female	19
*Picus*	*Picus viridis*	2	adult	female	20
*Picus*	*Picus viridis*	3	adult	male	21
*Picus*	*Picus viridis*	4	juvenile	female	22
*Picus*	*Picus viridis*	5	adult	male	23
*Picus*	*Picus viridis*	6	adult	female	24
*Picus*	*Picus viridis*	7	adult	male	25
*Picus*	*Picus viridis*	8	immature	male	26
*Picus*	*Picus viridis*	9	juvenile	male	27
*Picus*	*Picus viridis*	10	adult	female	28
*Picus*	*Picus viridis*	11	adult	female	29
*Picus*	*Picus viridis*	12	juvenile	female	30
*Picus*	*Picus viridis*	13	juvenile	female	31
*Picus*	*Picus viridis*	14	juvenile	male	32
*Picus*	*Picus viridis*	15	immature	female	33
*Picus*	*Picus viridis*	16	juvenile	female	34
*Picus*	*Picus viridis*	17	adult	female	35
*Dryocopus*	*Dryocopus martius*	1	adult	female	38
*Dendrocoptes*	*Dendrocoptes medius*	1	adult	female	37
*Dendrocopos*	*Dendrocopos major*	1	juvenile	female	1
*Dendrocopos*	*Dendrocopos major*	2	juvenile	male	2
*Dendrocopos*	*Dendrocopos major*	3	adult	male	3
*Dendrocopos*	*Dendrocopos major*	4	adult	male	4
*Dendrocopos*	*Dendrocopos major*	5	adult	female	5
*Dendrocopos*	*Dendrocopos major*	6	adult	male	6
*Dendrocopos*	*Dendrocopos major*	7	adult	male	7
*Dendrocopos*	*Dendrocopos major*	8	adult	male	8
*Dendrocopos*	*Dendrocopos major*	9	juvenile	male	9
*Dendrocopos*	*Dendrocopos major*	10	juvenile	female	10
*Dendrocopos*	*Dendrocopos major*	11	adult	male	11
*Dendrocopos*	*Dendrocopos major*	12	adult	male	12
*Dendrocopos*	*Dendrocopos major*	13	juvenile	female	13
*Dendrocopos*	*Dendrocopos major*	14	subadult	female	14
*Dendrocopos*	*Dendrocopos major*	15	juvenile	male	15
*Dendrocopos*	*Dendrocopos major*	16	juvenile	female	16
*Dendrocopos*	*Dendrocopos major*	17	juvenile	female	17
*Dendrocopos*	*Dendrocopos major*	18	adult	male	18
*Dryobates*	*Dryobates minor*	1	adult	male	39
*Dryobates*	*Dryobates minor*	2	adult	female	40

## Data Availability

The original contributions presented in this study are included in the article/Supplementary Material. Further inquiries can be directed to the corresponding authors.

## Supplementary Materials

The following supporting information can be downloaded at: https://www.mdpi.com/article/10.3390/vetsci13070702/s1, Figure S1. Molecular sex determination based on amplification of intron 16 of the CHD1 gene. Lanes 1–18: Great Spotted Woodpecker (*Dendrocopos major*); lanes 19–35: Eurasian Green Woodpecker (*Picus viridis*); lane 36: Gray-headed Woodpecker (*Picus canus*); lane 37: Middle Spotted Woodpecker (*Dendrocoptes medius*); lane 38: Black Woodpecker (*Dryocopus martius*); lanes 39–40: Lesser Spotted Woodpecker (*Dryobates minor*). The lane number corresponding to each examined individual is provided in Table 1. DL—GeneRuler 100 bp Plus DNA Ladder (Thermo Scientific, Waltham, MA, USA). Arrows indicate the sizes of the selected DNA ladder bands.
