# Supplementary material for: Histological Diversity of Eyelids and the Nictitating Membrane in Six Woodpecker Species (Picidae)
Source: Vet Sci. 2026 Jul 17;13(7):702. doi: 10.3390/vetsci13070702 (PMC13431540; doi:10.3390/vetsci13070702)
Supplement: Supplementary file 1 [file vetsci-13-00702-s001.zip › vetsci-4405897-supplementary.pdf]

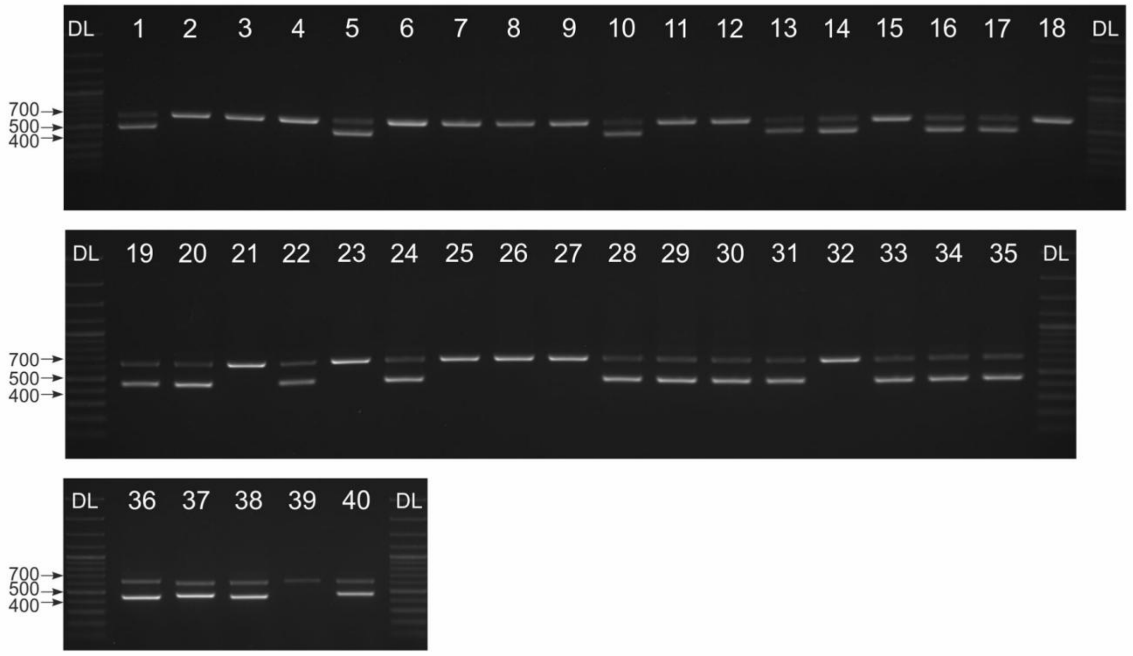

Figure S1. Molecular sex determination based on amplification of intron 16 of the CHD1 gene. Lanes 1–18: Great Spotted Woodpecker (*Dendrocopos major*); lanes 19–35: Eurasian Green Woodpecker (*Picus viridis*); lane 36: Gray-headed Woodpecker (*Picus canus*); lane 37: Middle Spotted Woodpecker (*Dendrocoptes medius*); lane 38: Black Woodpecker (*Dryocopus martius*); lanes 39–40: Lesser Spotted Woodpecker (*Dryobates minor*). The lane number corresponding to each examined individual is provided in Table 1. DL - GeneRuler 100 bp Plus DNA Ladder (Thermo Scientific). Arrows indicate the sizes of the selected DNA ladder bands.
